# Design and Experimental Analysis of Multiband Frequency Reconfigurable Antenna for 5G and Sub-6 GHz Wireless Communication

**DOI:** 10.3390/mi12010032

**Published:** 2020-12-30

**Authors:** Haris Dildar, Faisal Althobiani, Ikhlas Ahmad, Wasi Ur Rehman Khan, Sadiq Ullah, Naveed Mufti, Shakir Ullah, Fazal Muhammad, Muhammad Irfan, Adam Glowacz

**Affiliations:** 1 Department of Telecommunication Engineering, University of Engineering and Technology, Mardan 23200, Pakistan; haris8371@gmail.com (H.D.); ikhlasahmed725@gmail.com (I.A.); wasi.khan@uetmardan.edu.pk (W.U.R.K.); naveed@uetmardan.edu.pk (N.M.); shakirhayat.eng@gmail.com (S.U.); 2 Faculty of Maritime Studies, King Abdulaziz University, P.O. Box 80401, Jeddah 21589, Saudi Arabia; falthobiani@kau.edu.sa; 3 Department of Electrical Engineering, University of Engineering and Technology, Mardan 23200, Pakistan; 4 Electrical Engineering Department, College of Engineering, Najran University Saudi Arabia, Najran 61441, Saudi Arabia; 5 Department of Automatic Control and Robotics, Faculty of Electrical Engineering, Automatics, Computer Science and Biomedical Engineering, AGH University of Science and Technology, al. A. Mickiewicza 30, 30-059 Kraków, Poland

**Keywords:** reconfigurable antenna, Sub-6 GHz, 5G, IoT, smart cities

## Abstract

A low-profile frequency reconfigurable monopole antenna operating in the microwave frequency band is presented in this paper. The proposed structure is printed on Flame Retardant-4 (FR-4) substrate having relative permittivity of 4.3 and tangent loss of 0.025. Four pin diode switches are inserted between radiating patches for switching the various operating modes of an antenna. The proposed antenna operates in five modes, covering nine different bands by operating at single bands of 5 and 3.5 GHz in Mode 1 and Mode 2, dual bands (i.e., 2.6 and 6.5 GHz, 2.1 and 5.6 GHz) in Mode 3 and 4 and triple bands in Mode 5 (i.e., 1.8, 4.8, and 6.4 GHz). The Voltage Standing Waves Ratio (VSWR) of the presented antenna is less than 1.5 for all the operating bands. The efficiency of the designed antenna is 84 % and gain ranges from 1.2 to 3.6 dBi, respectively, at corresponding resonant frequencies. The achieve bandwidths at respective frequencies ranges from 10.5 to 28%. The proposed structure is modeled in Computer Simulation Technology microwave studio (CST MWS) and the simulated results are experimentally validated. Due to its reasonably small size and support for multiple wireless standards, the proposed antenna can be used in modern handheld fifth generation (5G) devices as well as Internet of Things (IoT) enabled systems in smart cities.

## 1. Introduction

The fifth generation (5G) of mobile communication operating in the sub-6 GHz frequency band is aimed for faster and reliable communication services with an enhanced network capacity. With the advancement of wireless communication technology requirement of multiple wireless services in a single device has increased significantly, so conventional antenna fails to meet this new requirement of wireless communication systems. To meet the demands, an antenna is designed which has an ability to switch its characteristics according to the requirements. Such an antenna is called a reconfigurable antenna [1]. Reconfigurable antennas are used for different wireless applications that operate in a wide range of frequency as they have proved to be very useful in completing new requirements of the system. Reconfigurable antennas can alter their behavior according to the requirements. They have ability to deliver the same performance as that of multiple antennas without increasing the size which would have occurred in case of using multiple antennas [2]. There are three basic types of reconfigurable antennas which include Frequency, Pattern, and polarization reconfigurable antennas. Frequency reconfigurable antennas provide frequency tuning over desired frequency bands and efficient utilization of spectrum [3]. Pattern reconfigurable antennas direct their radiation pattern towards a desired direction and are a fundamental concept for beam steering in the future mobile networks. Pattern reconfigurable antennas with beam reconfigurability can also be employed in Multiple-Input Multiple-Output (MIMO) services [4]. Polarization reconfigurable antennas reduce multipath fading, improve the effectiveness in receiving communication signal, and reduce co-channel interference [5].

The antenna can be made reconfigurable by using different types of switching techniques [6]. In [7], a liquid metal is used to obtain reconfigurability in a frequency-reconfigurable patch antenna for Industrial, Scientific, and Medical (ISM)/Global Positioning System (GPS) band. Radio frequency (RF) pin diodes are used in [8] to obtain reconfigurability for nine different frequency bands. Eight Varactor diodes are used for reconfigurability, which operates in the range of 1.64 GHz to 2.12 GHz in [9]. In [10], Radio Frequency Microelectromechanical Systems (RF-MEMS) switches are used for switching purposes at a faster rate with dual frequency reconfigurable bands (4.57 and 4.88 GHz) by integrating the antenna with a bias network. Electrically tuned plasma is used to achieve reconfigurability in low profile broad band plasma antenna for VHF and UHF applications [11]. In [12], a set of optical (photo conductive) switches are used for frequency and radiation reconfiguration with application of millimeter-wave (mmW) 28 GHz and 38 GHz frequency range. For beam steerable planer antenna, four PIN diodes is used as a switching device [13] to achieve reconfigurability for Worldwide Interoperability for Microwave Access (WiMAX) and Wireless Local Area Network (WLAN) applications. A reconfigurable MIMO antenna for cognitive radio applications is made with PIN and Varactor diodes reconfiguration mechanism in [14].

A CPW Fed Sub 6 GHz Frequency Reconfigurable Antenna for 5G and UWB Applications is presented in [15] where a single pin diode is used for reconfigurability. In [16] a Differentially-Fed Frequency Reconfigurable antenna is presented for WLAN and Sub-6GHz 5G Applications. These antennas can operate in either single or dual band modes depending on the state of switch. Lumped element switch is used to attain reconfiguration. In [17], author has presented an E-shaped frequency reconfigurable antenna in which Pin diodes, Varactor diodes and RF MEMS have been used to achieve reconfigurability. Circular and linear polarization occurs in the range of 2.4 to 3.6 GHz. A monopole frequency reconfigurable antenna is introduced in [18]. Three pin diodes are used for reconfigurability. There are four modes of operation. Each mode has different applications. Mode 1 for The Global System for Mobile Communications (GSM). Mode 2 for 3G advanced/Long Term Evolution (LTE). Mode 3 for WIFI/WLAN/ISM and Mode 4 is for Airport Surveillance Radar band/WLAN applications. In [19], a compact hexa-band frequency-reconfigurable antenna is presented. The proposed antenna operates at six different bands such as Wireless Fidelity (WI-FI), WI-MAX, Universal Mobile Telecommunications System (UMTS), and WLAN. 

In [20] Multiband frequency reconfigurable antenna is presented for 5G and Ultra-wideband (UWB) applications. A PIN Diode is employed to achieve frequency reconfiguration in the proposed antenna. A multi-band frequency reconfigurable Inverted F antenna for wireless applications is presented in [21]. An F-shaped frequency reconfigurable antenna is presented in [22] which covers different bands which include the WLAN, Wi-MAX, Wi-Fi, and GSM band. In [23] a tri-band frequency reconfigurable antenna is presented for LTE/WiFi)/ITS applications. Switching is done by two PIN diodes loaded on the ground plane. A multi band reconfigurable antenna for future wireless applications is presented in [24] in which total eight frequency bands between 1.46 and 6.15 GHz have been achieved using two pin diodes. A compact multiband frequency reconfigurable planar inverted-F antenna (PIFA) is presented in [25]. Seven different bands have been achieved by using a single RF switch for switching mechanism. The antenna is designed for GPS, LTE, UWB and satellite applications. In [26] a multi-band frequency reconfigurable antenna for 5G communication is reported. Two pin diodes have been inserted in the triangular-shaped radiator for switching the modes. Antenna has two operational modes covering five resonant bands. In [27], a dipole-based frequency reconfigurable antenna is presented, having four pin diodes for switching. Depending upon switching states of pin diodes antenna has two resonant modes, covering three different bands for sub-6GHz and WLAN applications. In [28], a composite right/left-handed (CRLH) unit cell loaded frequency reconfigurable antenna is reported. Antenna has two varactor diodes with varying the capacitance providing ten different LTE bands. In [29] a compact CPW fed frequency reconfigurable antenna is presented. Two pin diodes have been used for switching, providing dual and tri-band operational modes. Similarly, a CPW fed multi-mode frequency reconfigurable antenna for Sub-6GHz and other wireless applications is reported in [30]. Three pin diodes have been loaded between stubs to achieve reconfigurability. Antenna has a wide band and four reconfigurable bands.

A novel shaped frequency reconfigurable multi band antenna designed on an FR-4 substrate is presented in this paper. The proposed antenna is reconfigurable using pin-diode switches and radiates on nine different bands with promising gain, radiation efficiency and compact size. The proposed antenna contains four pin diode switches. 

This paper is organized as follows: Section 2 explains the design methodology and geometry of the proposed switchable multiband antenna. The analysis of simulation and measured results is discussed in Section 3 and Section 4 concludes this research work.

## 2. Methodology

This section presents basic geometry and design theory of the proposed multi band frequency reconfigurable antenna. Frequency reconfigurability is achieved in simulation through the lumped element RLC equivalent circuit of the pin diodes. The designed antenna can be operated in different frequencies by using the ON/OFF condition of the diodes. Better efficiency and satisfactory far field results have been obtained using a partial ground plane.

### 2.1. Design Theory and Structural Geometry

**Figure 1:**
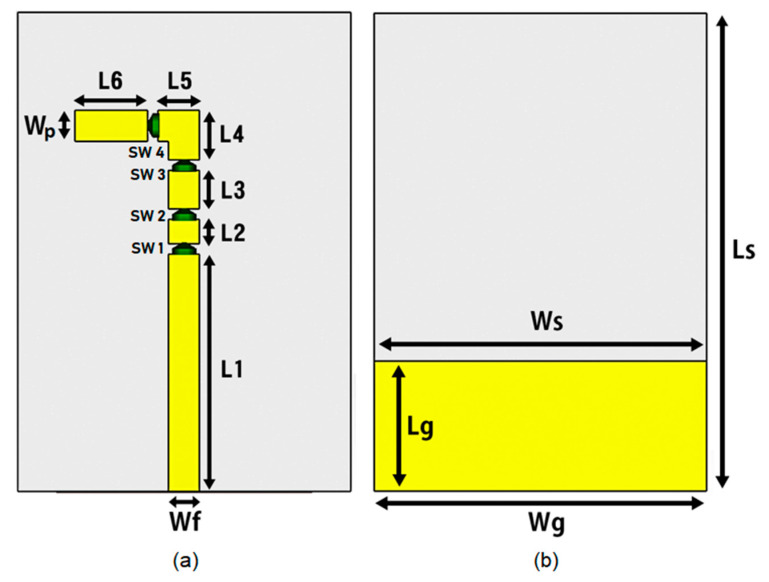
The geometry of proposed antenna: (**a**) Front view (**b**) Rear view.

The geometrical structure of the proposed antenna is depicted in Figure 1. The presented antenna of dimension 40 × 32 × 1.6 mm^3^ which is based on a 1.6 mm thick FR-4 substrate. The substrate has relative permittivity $\varepsilon_{r}$ of 4.3 and loss tangent δ of 0.025, backed by truncated metallic ground plane to obtain better gain, good efficiency, and directivity. For insertion of the pin diode switches, a gap of 1 mm was kept between the patches. A microstrip line of width 3 mm having 50 Ω impedance is used to excite the antenna. 

**Figure 2:**
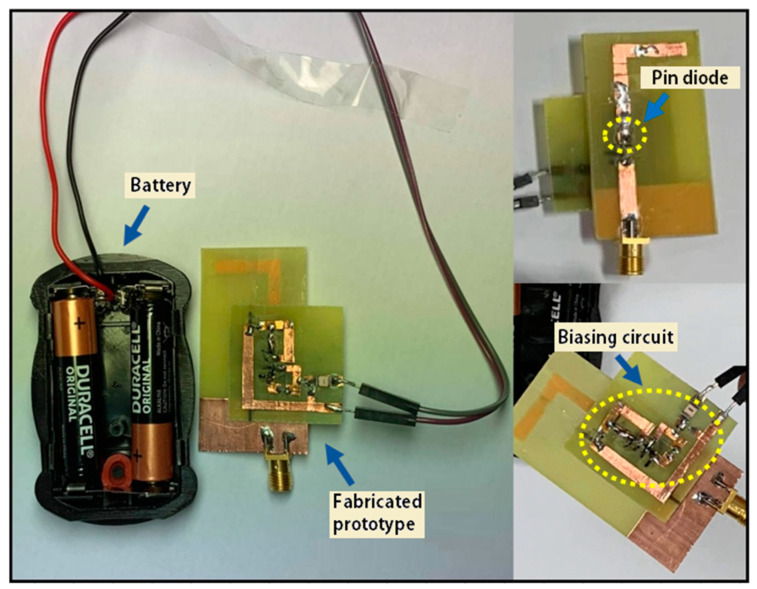
The view of fabricated prototype with biasing circuit.

Pin diode (SMP1345-079LF) is used for switching in the simulations. The biasing circuit for operating the pin diode switch is designed on FR4 substrate. The configuration to connect the biasing circuit to control the pin diode, implanted on the fabricated prototype, is portrayed in Figure 2. For the proof of concept, only a single switch (pin diode) is operated through the biasing circuit. The remaining three switches were turned ON by soldering copper strips across the switch position, for a particular frequency mode (i.e., mode 3, 4, and 5).

A detailed summary of the dimensions of the proposed antenna is presented in Table 1. 

The effective resonant lengths for intended frequencies are calculated using transmission line model theory [31]. The effective length of the antenna corresponding to the respective resonant bands are one quarter of the guided wavelength (i.e., *L_f_* = *λ_f_*/4). 

### 2.2. Reconfigurability

In proposed antenna frequency reconfigurability is achieved by changing the ON and OFF states of each PIN diode that offers an open and short circuit behavior between radiating patches. The presented antenna has five operating modes, each have unique scheme of resonant frequencies. In Mode 1 (SW1 to SW4 = OFF), antenna operates at 5 GHz. For Mode 2 (SW1 = ON, SW2 to SW4 = OFF), the proposed antenna resonates at 3.5 GHz. The antenna shows dual band behavior and covers 2.6 and 6.5 GHz at Mode 3 (SW1 and SW2 = ON, SW3 and SW4 = OFF). The same antenna covers two different bands of 2.1 and 5.6 GHz when operating at Mode 4 (SW1 to SW3 = ON, SW4 = OFF). The three unique bands of 1.8, 4.8, and 6.4 GHz are covered when antenna operates in Mode 5 (SW1 to SW4 = ON). The conditions of the PIN diodes at each mode and respective resonant bands are detailed in Table 2.

### 2.3. Switching Technique

**Figure 3:**
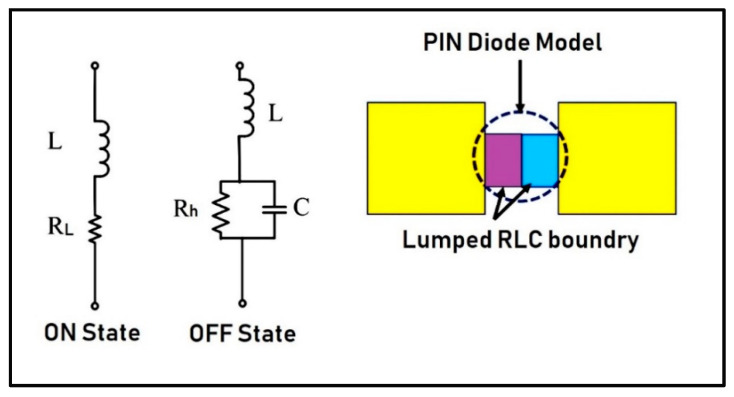
PIN diode model and its Equivalent circuits for ON and OFF states.

For switching purpose four pin diodes (SMP1345-079LF) are used, as they behave like a variable resistor in the radio frequency (RF) range. These pin diodes provide open and the short circuit behavior at their respective insertion positions, thus vary the effective resonant length of the antenna and hence result in reconfiguration of antenna’s operating frequency. The equivalent circuits for both ON and OFF states of a pin diode switches are shown in the Figure 3. For ON state it is simply an RL series circuit, having a low value resistor “$R_{L}$” and an inductor “*L*”. In OFF state it is equivalent to an RLC circuit, having inductor “*L*” in parallel with a high value resistor “$R_{H}$” and a capacitor “*C*”. Pin diode of model Skyworks SMP1345-079LF are used in this work. According to its datasheet it has been modeled in CST as *R_L_* = 1.5 Ω, *L* = 0.7 nH and *C* = 0.15 pF.

## 3. Results and Discussion

**Figure 4:**
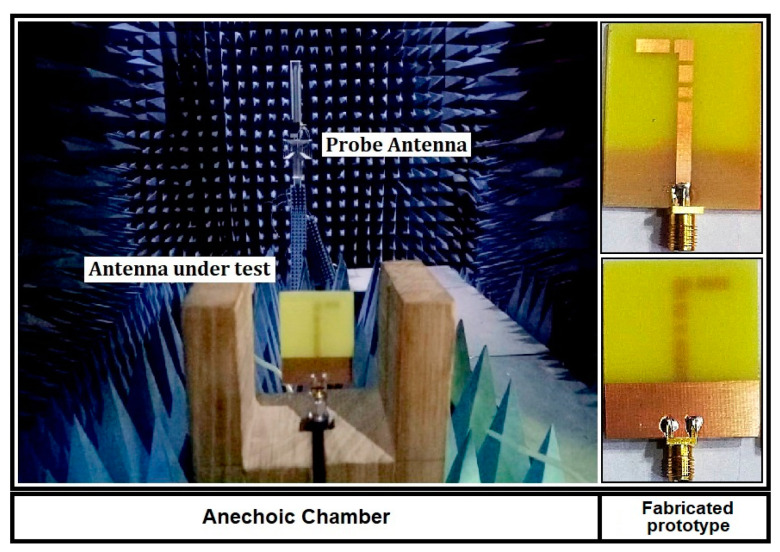
Setup for measuring radiation pattern of the proposed antenna in Mode 1.

The proposed structure is designed and analyzed using CST microwave studio. To excite the radiating structure, a waveguide port of standard dimensions is assigned. The performance parameters i.e., return loss, gain, surface current plots are obtained by using the standard boundary conditions in CST microwave studio. The simulated results are experimentally validated in the antenna measurement facility located at National University of Science and Technology (NUST) Islamabad. The experimental setup to measure the radiation pattern of the proposed antenna in mode 1 is depicted in Figure 4. 

### 3.1. Return Loss and Bandwidth

**Figure 5:**
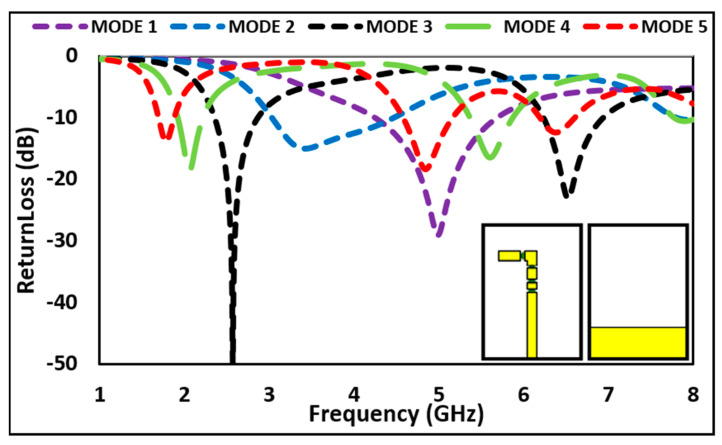
Return loss for all operating modes.

The simulated return loss of all modes of the proposed antenna are depicted in Figure 5. When all switches (SW1 to SW4) are OFF, the proposed antenna operates in MODE 1, resonating at 5 GHz with return loss of −29.33 dB and simulated bandwidth of 1400 MHz (4.21–5.61 GHz). In MODE 2 (When SW 1 is ON), the presented antenna resonates at 3.5 GHz with return loss of −15.17 dB and bandwidth of 1370 MHz (3.03–4.42 GHz). In MODE 3 when two switches (SW1, SW2) are ON and other two switches (SW3, SW4) are OFF, the proposed antenna operates at two different bands, i.e., 2.6 and 6.5 GHz with −50 dB and −23.65 dB return loss and bandwidth of 500 MHz (2.40–2.90 GHz) and 670 MHz (6.27–6.94 GHz), respectively. The same antenna covers two different bands of 2.1 and 5.6 GHz when SWI, SW2 and SW3 are ON and SW4 is OFF, with return loss of −18 dB and −16.36 dB and bandwidth of 300 MHz (1.95–2.25 GHz) and 450 MHz (5.41–5.86 GHz), respectively at the operating frequencies. When all switches (SW1 to SW4) are ON, the antenna switch to its MODE 5 and operates at 1.8, 4.8, and 6.4 GHz with return loss of −13.76 dB, −18.67 dB, and −13.6 dB and bandwidth of 190 MHz (1.71–1.90 GHz), 580 MHz (4.55–5.13 GHz), and 450 MHz (6.18–6.63 GHz), respectively. 

**Figure 6:**
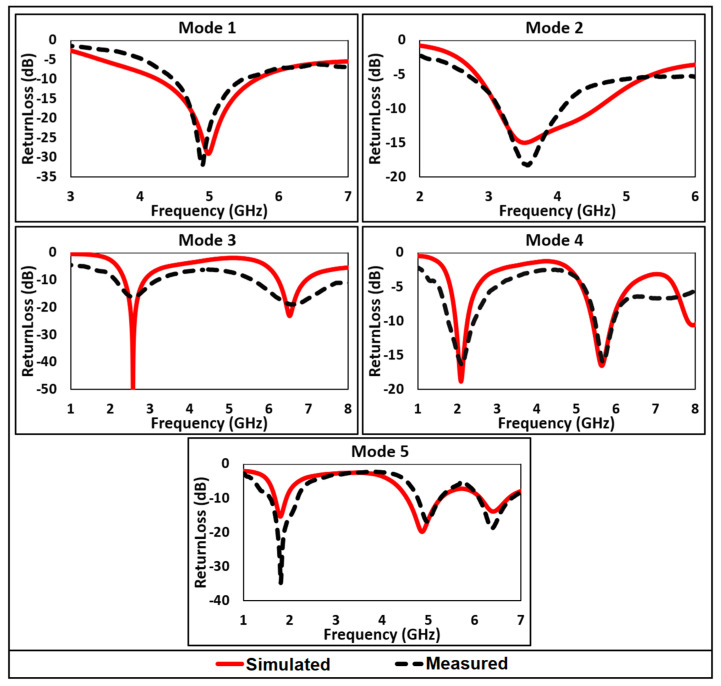
Comparison of simulated and measured return loss.

**Figure 7:**
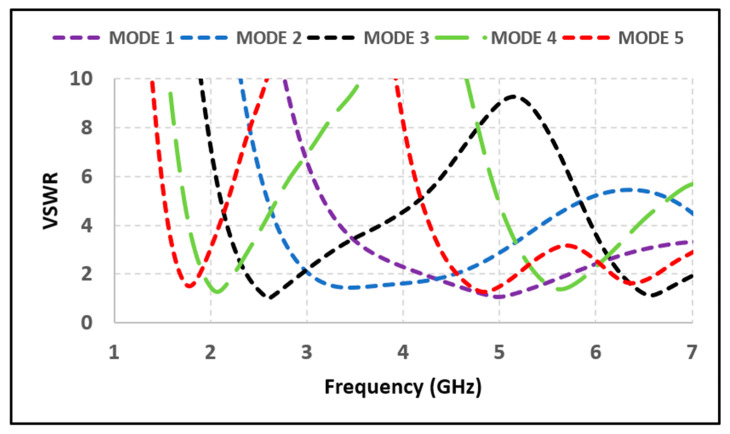
Voltage Standing Waves Ratio (VSWR) of proposed antenna at various operating modes.

The simulated and measured return loss of each mode is compared in Figure 6. The comparison shows a good agreement between measured and simulated results. The Voltage Standing Waves Ratio (VSWR) of less than 1.5 is observed for all resonant bands, which indicates optimum driving-point impedance matching of the antenna. The VSWR is less than 2 for all the operating frequency bands, as depicted in Figure 7.

### 3.2. Far Field Radiation Pattern

In MODE 1, proposed antenna operates at 5 GHz with a simulated peak gain and radiation efficiency of 1.25 dBi and 81%, respectively. In MODE 2, a central frequency of 3.5 GHz has been achieved with peak gain and radiation efficiency of 2.17 dBi and 82 %, respectively. A dual band is achieved in MODE 3 with gain of 1.76 and 1.9 dBi and radiation efficiencies of 84% and 71% at 2.6 and 6.5 GHz, respectively. The antenna operates at 2.1 and 5.6 GHz in MODE 4, with peak gains of 1.4 and 2.15 dBi and radiation efficiencies of 83% and 70%, respectively. In MODE 5 the proposed structure operates at 1.8, 4.8 and 6.4 GHz, with peak gains of 1.2, 2.37, and 3.6 dBi and radiation efficiencies of 82%, 75%, and 74%, respectively. 

**Figure 8:**
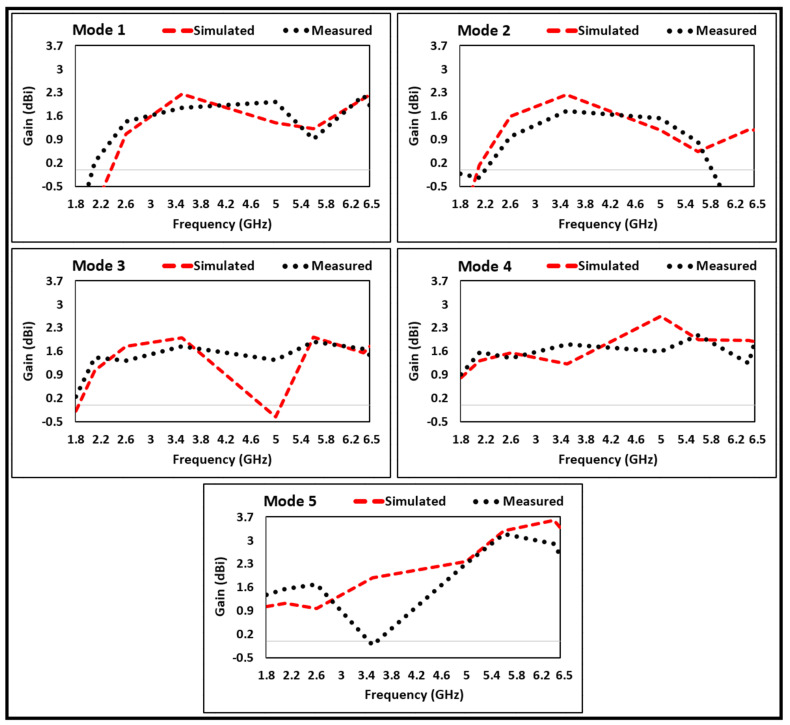
Comparison of simulated and measured gain.

The comparison of simulated and measured gain of proposed antenna is depicted in Figure 8 showing that the measured gains are in a good agreement with the simulated ones. It is worth mentioning that the antenna gives optimum values of average gain, i.e., 1.1 dBi in mode 1, 1.98 dBi in mode 2, 1.69–1.8 dBi in mode 3, 1.28–2 dBi in mode 4, and 1.12–3.45 dBi in mode 5.

**Figure 9:**
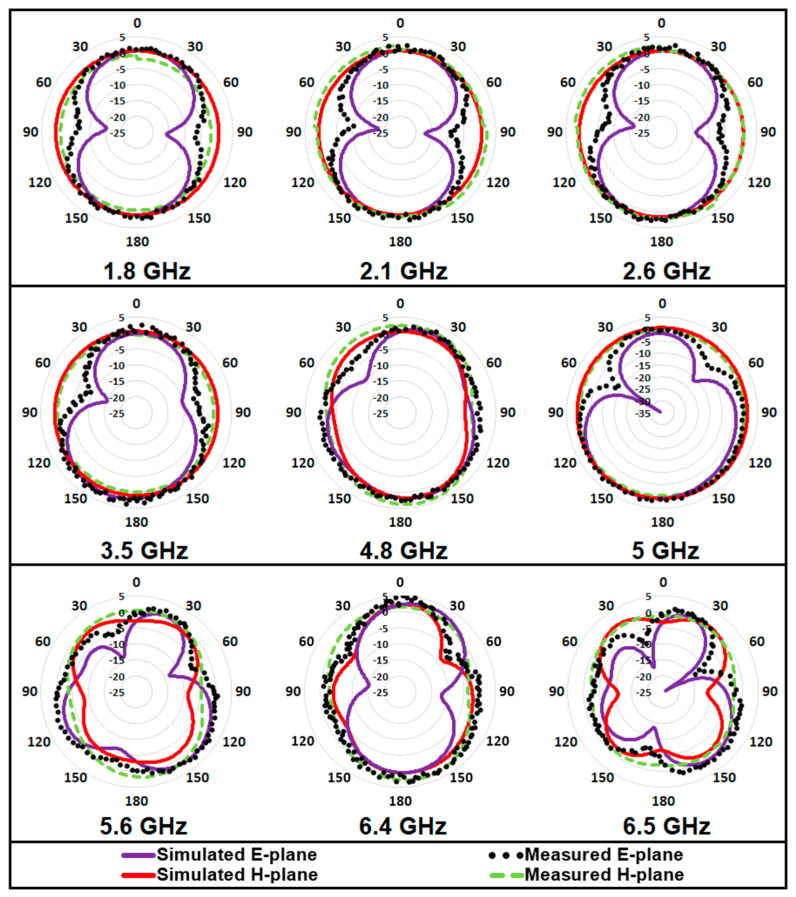
Comparison of simulated and measured radiation pattern in both principal planes.

The simulated and measured radiation pattern of the antenna in both E- and H- plane at the operating frequency bands, are compared in Figure 9. The shape of the radiation pattern in the E-plane, resembles the figure-of-eight at frequencies of 1.8, 2.1, 2.6, 3.5, and 6.4 GHz. The radiation properties of the antenna in the H-plane are predominantly Omni-directional in nature in most of the frequency bands. The half power beamwidth (HPBW) and main lobe direction (MLD) were deduced from the E-plane radiation pattern of the antenna in all resonant modes, and the same are summarized in Table 3. 

**Figure 10:**
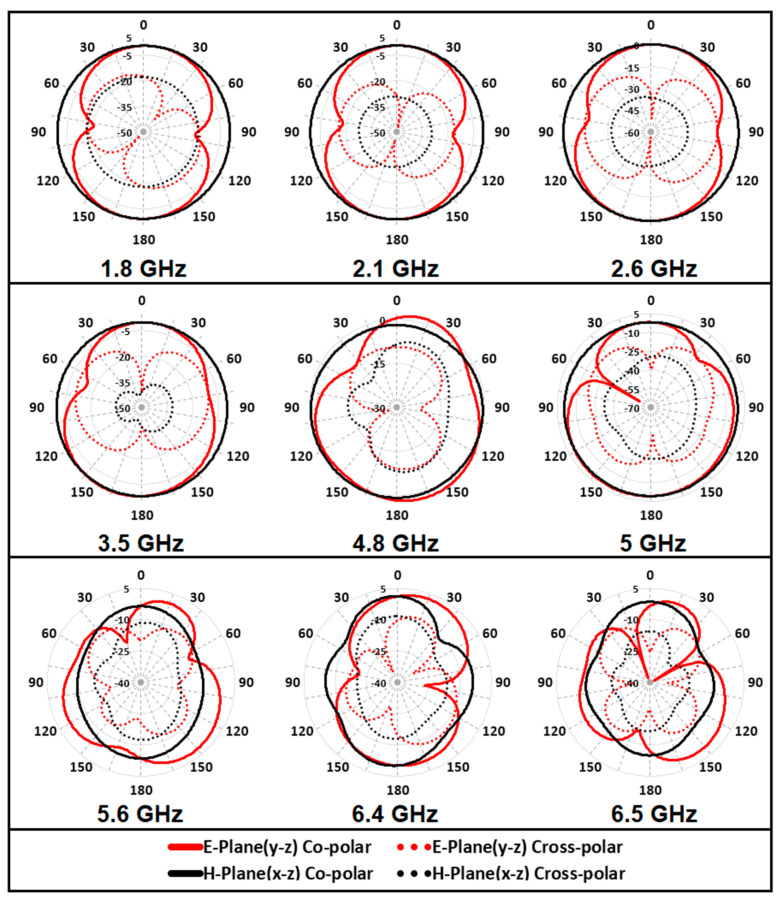
Co- and cross-polar radiation pattern for both principal planes.

**Figure 11:**
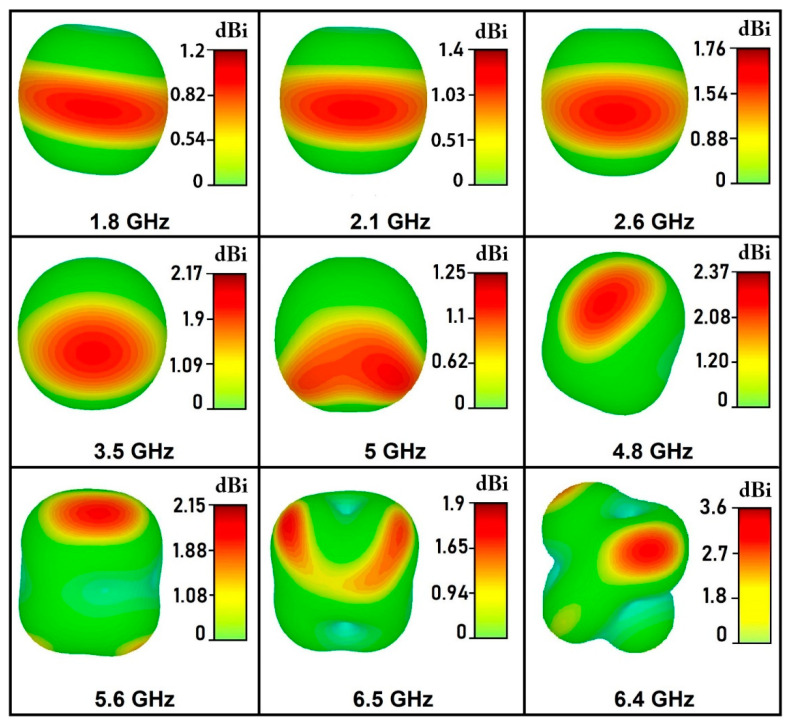
The three-dimensional plots of gain pattern

The simulated co- and cross-polar radiation patterns for both E-plane (y-z) and H-plane (x-z) are presented in Figure 10. It is evident from the results that in the co-polarization state, the antenna radiates adequately in both E- and H-planes. While, in cross-polarization state, the antenna gain is predominately negative and hence the radiation is extremely poor in both principal planes. For further clarity about the radiation properties of the antenna, the three-dimensional gain plots are portrayed at the resonant frequencies in Figure 11.

### 3.3. Surface Currents

**Figure 12:**
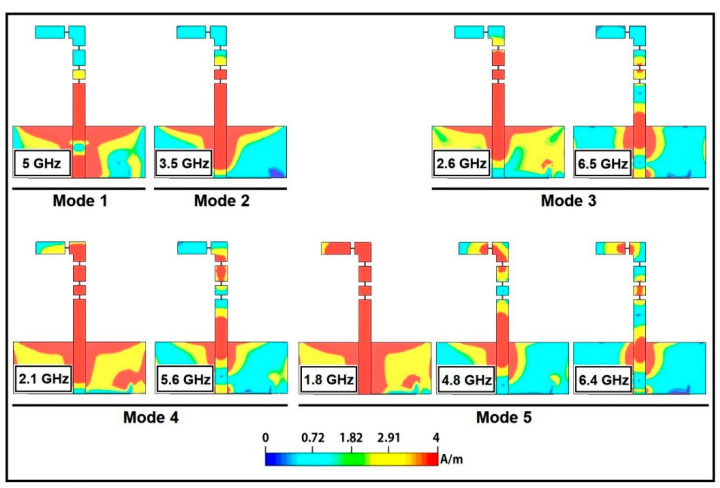
Surface currents plots of antenna at various modes.

The surface current distribution on the radiating structure of the antenna at different frequency bands is shown in Figure 12. For Mode 1, antenna operates at 5 GHz frequency, the density of the surface currents is higher on the main radiator which is contributing in radiation at 5 GHz. In Mode 2, the surface currents indicate an increase in the contributing resonant length, thus operation shifts to 3.5 GHz. In Mode 3 and 4, dual band operations (i.e., 2.6 and 3.5 GHz, 2.1 and 5.6 GHz) are achieved, the surface currents indicate the dominant contribution of larger portion of radiator for lower bands and smaller portion of radiator for the upper bands. In Mode 5, triple band (i.e., 1.8, 4.8, and 6.4 GHz) operation is achieved, the higher surface current density along the entire length of the radiator indicate that the whole metallic radiator is contributing in radiation in the lower frequency band (i.e., 1.8 GHz). It is worth mentioning that relatively smaller segments of the radiator are contributing in radiation at the upper bands (4.8 and 6.4 GHz). These surface currents indicate that the contributing resonant length for respective frequency decreases as resonant frequency increases, thus proves the inverse relation of frequency with resonant length. Performance matrices of proposed antenna are summarized in Table 4. The power consumption by the parasitic resistance of the pin diodes and as well as the power loss in the dielectric substrate were analyzed for each operating frequency. The average power consumption or loss contributed by the pin diodes and dielectric substrate is 0.023 w and 0.031 w, respectively.

In terms of size, the proposed antenna is more compact than other antennas reported in [32,33,34,35,36,37,38]. The proposed antenna has impedance bandwidth ranges 190–1400 MHz which offers larger bandwidth than antennas designed in [32,33,35,36,37,38,39] and has better gain than the work reported in [32,33,34,36,37,39]. The most prominent distinction, as compared to other candidate antennas, is the ability of proposed antenna to operate on nine different frequency bands, which are more than the number of bands achieved in all reported works. The comparison is given in Table 5. 

## 4. Conclusions

A frequency reconfigurable antenna has been designed, simulated, and experimentally validated in this paper. The proposed antenna is reconfigured depending on the ON and OFF states of the pin diode switches. When all the switches are OFF, the presented antenna covers a single band (5 GHz). Antenna operates at 3.5 GHz when only SW1 is ON. Dual bands i.e., 2.6 GHz and 6.5 GHz are achieved when SW1 and SW2 are ON. The prototype works in dual band mode (2.1 GHz and 5.6 GHz) when SW1 to SW3 are ON. When all switches are ON, the designed antenna operates in triple band (1.8, 4.8, and 6.4 GHz). The proposed antenna has many advantages like compact size, low cost, light weight and easy of fabrication and supports sub-6GHz 5G bands (2.1, 2.6, 3.5, and 4.8 GHz) with an extended application for GSM, UMTS, 4G-LTE, WiMAX, WLAN Wireless networks, Internet of Things (IoT) enabled wireless systems in smart cities as well as the 6 GHz Fixed Satellite Services.

## Figures and Tables

**Table 1:** Dimensions of proposed antenna.

Parameters	Values (mm)	Parameters	Values (mm)
Ws	32	L2	2.3
Ls	40	L3	3.7
Wg	32	L4	4.8
Lg	12.5	L5	4
Wf	3	L6	7
L1	22.8	Wp	3

**Table 2:** Conditions of the PIN diodes for various resonant bands.

MODES	SW1	SW2	SW3	SW4	Resonant Bands (GHz)
1	OFF	OFF	OFF	OFF	5
2	ON	OFF	OFF	OFF	3.5
3	ON	ON	OFF	OFF	2.6 and 6.5
4	ON	ON	ON	OFF	2.1 and 5.6
5	ON	ON	ON	ON	1.8, 4.8 and 6.4

**Table 3:** Summary of half power beamwidth and main lobe direction.

Mode No	Operating Frequency	HPBW (in Degrees)	MLD (in Degrees)
1	5	121	−155
2	3.5	99.2	−165
3	2.6/6.5	90.8/60	−170/145
4	2.1/5.6	90/69	−175/140
5	1.8/4.8/6.4	90/106/51	−175/160/5

**Table 4:** Summary of the antenna’s performance.

Mode No	Conditions	Operating Freq (GHz)	−10 dB BW (MHz)	Gain (dBi)	Return Loss (dB)	Radiation Efficiency (%)
1	When all switches are OFF	5	4.21–5.61 (1400)	1.25	−29.33	81
2	When S1 is ON	3.5	3.05–4.42 (1370)	2.17	−15.17	82
3	When S1 and S2 are ON	2.6/6.5	2.40–2.90 (500)/6.27–6.94 (670)	1.76/1.9	−50/−23.65	84/71
4	When S1, S2 an S3 are ON	2.1/5.6	1.95–2.25 (300)/5.41–5.86 (450)	1.4/2.15	−18/−16.36	83/70
5	When S1, S2, S3 and S4 are ON	1.8/4.8/6.4	1.71–1.90 (190)/4.55–5.13 (580)/6.18–6.63 (450)	1.2/2.37/3.6	−13.76/−18.6/−13.6	82/75/74

**Table 5:** Proposed antenna’s comparison with other reported works.

Ref.	Dimensions (mm^3^)	Total No of Operating Bands	Operating Frequencies (GHz)	Bandwidth (MHz)	Peak Gains (dBi)	Radiation Efficiency (%)
[32]	40 × 35 × 1.6	3	2.45, 3.5, 5.4	490–1360	1.92–3.02	76.4–86.5
[33]	60 × 60 × 1.6	5	2.4, 4.26, 4.32, 4.58, 5.76	60–170	1.31–2.77	-
[34]	53 × 35 × 1.6	3	2.45, 3.50, 5.20	147–1820	1.7–3.4	85–90
[39]	40 × 22 × 1.6	4	2.45, 5.13, 3.49, 5.81	750–1260	1.72–2.96	76.4–92
[35]	39 × 37 × 1.6	3	2.4, 5.4, 3	550–1220	1.27–3.8	>90
[36]	37 × 35 × 1.6	4	2, 3.4, 2.4, 3.1	200–960	1.76–1.98	>85
[37]	40 × 35× 1.6	3	2.45, 3.5, 5.2	330–1250	1.48–3.26	84–93.5
[38]	40 × 35 × 1.6	6	2.10, 2.40, 3.35, 3.50, 5.28, 5.97	335–1220	1.92–3.8	92.5–97
This work	40 × 32 × 1.6	9	1.8, 2.1, 2.6, 3.5, 4.8, 5.0, 5.6, 6.4, 6.5	190–1400	1.25–3.6	70–84
